# Chemistry of the Cinchona Barks, (Cincho-Quinine.)

**Published:** 1869-06

**Authors:** 


### Chemistry of the Cinchona Barks, (Cincho-Quinine.)

The chemical manipulation of the Cinchona or Peruvian Barks reveals the presence in them of quite a number of most remarkable, complex bodies. No vegetable production, except the poppy, affords such a marvelous combination of valuable medicinal principles as the *loxa* and *calisaya* barks, and no substances have been studied with greater care or more intense interest by chemists. Nothing short of the subtle chemical forces controlled by the Infinite One could construct from the elements of the earth and air a bitter principle like quinia, or those other agents associated in bark, so closely allied to it physically and chemically. A handful of the finely comminuted fibres of the yellow bark, which resemble physically a dozen other varieties, is made to yield by the chemist, when treated with aqueous and alcoholic liquids and acids, a dark, bitter solution, unattractive in taste and appearance. If the process is skillfully conducted, or exhaustive in its results, there remains, beside the solution, a portion of woody fibre, inert and almost tasteless. It holds considerable coloring and some waxy matter, together with a little tannin; but the active chemical or medicinal principles have been removed, and are held in the dark liquid. The exhausted bark is not entirely worthless, for it may be dried and used as fuel. But what of the dark liquid? From this the chemist obtains, beside other substances, a portion of beautiful, white, silky crystals; not wholly of one distinct kind, but of several, all of which possess about equal chemical and therapeutical importance. No wonder it seems, to the uninitiated in chemical manipulation, a difficult work to perform. It is, however, quite easy to the thoroughly instructed. The first principle isolated may be the quinia. This is not held in the bark in its naked alkaloidal condition, but locked up, in the form of a salt, with another principle called *kinic acid.* In the bark it is *kinate of quinine.* We isolate the quinia, tear it from its embrace with kinic acid, throw that away, force it into a kind of matrimonial alliance with sulphuric acid, and in this condition of *sulphate of quinia* use it as a medicine. This kinic acid marries into several other families resident in the bark, prominent among which are *cinchonia*, *cinchonidia*, *quinidia*, etc. Precisely how many of these alkaloidal principles the different kinds of barks contain, is unknown; but it is safe to assume that there are as many as four others which, although not distinctly pointed out, are tolerably well recognized. These *kinates* are all *kindred* in nature, and all labor to the same end, when isolated and set to work as therapeutical agents in the human system.

In one hundred ounces of good yellow bark, we obtain about two and three-fourths ounces of quinia, and two ounces of cinchona, with variable amounts of the other principles, but less than the two named. It is to be regretted that we cannot remove the different families of kinates from the bark in their natural state of saline combination. It seems reasonable to suppose their action upon the system would be more salutary than in other forms. It is easy to isolate the kinic acid, and these having the alkaloids, the kinates of quinia, cinchonia, etc., can be reformed; but in these chemical changes so much disturbance to natural organic combinations is made, that, practically, we realize no marked advantages. It seems unnatural to force a natural alkaloidal base out of its association with an organic acid, and recombine it with a mineral acid. This we do in the preparation of the sulphate of quinia. However, as it has served so good purpose for many years, it is not best to quarrel with the theory.

All the alkaloids of bark possess about equal febrifuge and tonic properties, when isolated and administered in that condition. This has been proved over and over again by all competent chemists and physicians, from Drs. Gomez, Duncan, Pelletier, Caventou, down to the time of Liebig's researches, a quarter of a century ago, and from that time to the present by a hundred careful chemical and medical observers.

How the one alkaloid, quinia, came to supercede the others, and drive them into the background, is easily understood, when we remember that it was about the first that was distinctly eliminated, studied, and experimented with; and the *eclat* it acquired caused everything else to be neglected. The natural bark, holding all the alkaloids, the quinia, cinchonia, quinidia, etc., has always been observed to produce more efficient and prompt results, both as a tonic and febrifuge, than the quinia, or either of the other principles in themselves; but holding also, as it does, tannin, gum, starch, fibrine, and coloring matter, all of which are medicinally interfering or inert, its use is rendered inconvenient and inadmissible in many cases. Beside, it is apt to produce disturbance of the gastric functions of an unpleasant character. Acting upon the idea that the natural alkaloidal principles of bark, in their simple, unchanged condition, separated from the gross, woody, and other matters, would better subserve all therapeutical ends than the barks themselves, or *any one* of the alkaloids separately employed, we have prepared CINCHO-QUININE.

Cincho-quinine contains no external agents, as sugar, licorice, starch, magnesia, etc. *It is wholly composed of the bark alkaloids.* 1st, quinia; 2d, cinchona; 3d, quinidia; 4th, cinchonidia; 5th, other alkaloidal principles present in barks, which have not been distinctly isolated, and the precise nature of which are not well understood. In the beautiful, white amorphous scales of cincho-quinine, the whole of the active febrifuge and tonic principles of the cinchona barks are secured without the inert, bulky lignin gum, etc. It is believed to have these advantages over sulphate of quinine: 1st, it suits the patient better; 2d, is not unpleasantly bitter; 3d, is much cheaper; 4th, it meets indications not met by that salt.

Cincho-quinine admits of several pleasant forms of administration. 1st. It may be given in pill form, mixed up with some pleasant, inert substance. 2d. It may be spread upon bread and butter, and taken in that way. 3d. It may be given suspended in milk or syrups, or in sweet wine, mixed with sugar. 4th. When it is taken in one or two grain doses as a simple tonic, most patients will not object to placing it upon the tongue and swallowing it with the saliva. Its action will, in many cases, be promoted by using in connection a glass of weak lemonade, sour wine, or some slightly acid drink. If mixed with acid wines, or sour liquids of any kind, a great increase in bitterness is developed, not enough, however, to render it unpleasant to most patients. If eight or ten grains are placed in a glass of water, and a few drops of acetic acid added, a clear solution is produced, which is very bitter. It is doubtful if it can be placed in a form suited to hypodermic use. But few experiments have, however, been made in this direction.

In intermittents, cincho-quinine may be given in 5, 10, 20, or even 30 grain doses, the same as sulphate of quinine. As an introduction to the treatment of fever and ague with cincho-quinine, an ipecac, or other emetic, is often of the greatest service. Ten or twenty grains of cincho-quinine, or of sulphate of quinine, might as well, so far as medicinal effect is concerned, be dissolved in the contents of a waste bucket, as in a stomach loaded with food and the attendant juices. The remedy must act upon the walls of the stomach and the connecting organs, to produce constitutional effects.

In a recent visit to the wards of the United States Marine Hospital, Chelsea, Dr. Graves, the physician in charge, politely invited us to thoroughly examine the numerous intermittent patients, with reference to his treatment of this disease, it being based upon the use of emetics prior to administering the bark preparations. We believe the wards of no hospital in the world can show more cases of prompt and thorough recovery from the affection than this. It is not probable that the marked beneficial influence of the emetic is due alone to its work as an evacuator, but its general influence on the system admirably prepares it for the use of antiperiodics.—*Boston Journal of Chemistry.*

